# A step-by-step guide for remote working in the NHS: evaluation of a virtual consultant psychiatrist hiring scheme

**DOI:** 10.1192/bjb.2024.56

**Published:** 2025-04

**Authors:** Megan Havard, Nyembezi Faith Ndebele, Suyog Dhakras, Gemma Johns, Ian McCafferty, Alka Ahuja

**Affiliations:** 1 TEC Cymru, Aneurin Bevan University Health Board, Newport, UK; 2 Solent NHS Trust, Southampton, UK

**Keywords:** Clinical governance, qualitative research, health informatics, quality improvement, community mental health teams

## Abstract

**Aims and method:**

In 2021, Solent NHS Trust advertised for a fully remote consultant psychiatrist to meet increasing clinical demand. This pilot scheme was evaluated to determine its success. The job applications underwent content analysis, recruitment and support staff were interviewed, and in-depth rolling interviews were conducted with the three now-employed virtual psychiatrists.

**Results:**

We have gained an objective understanding of this new and innovative way of working and, overall, shown that fully remote working in the National Health Service (NHS) is feasible.

**Implications:**

The findings were used to create a step-by-step guide for the remote hiring process, which outlines the necessary steps for conducting it in a safe, swift and successful way. This guide could help other NHS organisations to advertise, recruit and manage fully remote employees.

Before the COVID-19 pandemic, virtual working was not as prevalent or widely accepted in the National Health Service (NHS) as it is today,^1^ owing to the assumption that healthcare roles require significant face-to-face interaction. The pandemic, however, challenged this belief, as lockdown restrictions forced clinics to adopt entirely remote delivery.^2^ Post-pandemic, virtual consultations and at-home virtual working for healthcare professionals have become more commonplace.^3^ Consequently, most NHS trusts and health boards are now advertising more for hybrid options, usually described as ‘flexible’ or ‘home working’.^4^ As most of these roles require some in-person contact, the applicant must live near the relevant NHS site. Fully remote working addresses this geographical issue, which can be especially beneficial for both staff and patients in rural areas of the UK^5^ and for the approximately one-third of NHS doctors who are international.^6^ It can provide the flexibility for these individuals to work from their home country some or all of the time, allowing them to be closer to family and fulfil care responsibilities and enhancing their work–life balance. These workers can help to alleviate the NHS's struggle to recruit and retain staff amid issues such as burnout,^7^ while also contributing to communities and healthcare in their own countries.^8^ Furthermore, there is a disparity between the cost of living in the UK and NHS pay,^9^ but in many other countries, a UK salary is considered to provide a high standard of living, making remote work in the NHS an appealing opportunity.

## Background of initiative

In 2021, Solent NHS Trust reflected on the ongoing regional problem to fill and sustain traditional, in-person posts for consultant psychiatrists in adult services. Neighbouring trusts also had many vacant positions, necessitating the exploration of new ways of working to meet the increasing clinical demand.

Remote working and telepsychiatry (delivery of psychiatric services at a distance via telecommunications) have proven successful and safe during the pandemic, as noted by Solent NHS Trust and documented in the literature.^10-14^ In fact, the use of telepsychiatry pre-dates the pandemic, having long been demonstrated to be an effective option that is received positively by both patients and clinicians.^15-21^ The continued use of telepsychiatry has been encouraged post-pandemic, with the pandemic having acted as a catalyst for this important digital movement.^13,22^

Eager to incorporate remote working as a long-term recruitment strategy, Solent NHS Trust decided to advertise for a 100% virtual consultant psychiatrist (VCP) position, significantly broadening the pool of eligible applicants. Having initially planned to hire one VCP, the overwhelmingly positive response led to the decision to hire three. This enabled more tailored roles based on applicants’ interests and was deemed more cost-effective than hiring locums. The VCPs are now permanently employed by Solent NHS Trust, virtually managing patients with all the conditions that would typically be seen in an adult community mental health setting, including neurotic, stress-related and somatoform disorders, personality disorders, mood disorders, schizophrenia and other psychotic disorders. The setup garnered interest from other boards and medical directors, prompting the creation of this step-by-step guide to aid employers in advertising, recruiting and managing fully remote workers from the UK and abroad. To our knowledge, this is the first documented description of how to recruit, onboard and manage fully virtual workers in the UK NHS.

## Method

A content analysis was performed on the 16 anonymised job applications shortlisted for the VCP position, to understand what types of professional applied for this role. This process involved familiarisation with the written text, before identifying codes and categories that corresponded throughout. The data were then analysed in more detail against these codes, with frequencies calculated to identify the most common themes. Posts on Twitter responding to the job advertisement were also analysed to gain insight into perceptions of the advert on social media. It should be noted, however, that those who decided to post on their social media in response do not represent the opinions of everyone who saw the advertisement.

Next, semi-structured interviews were conducted with recruitment and support staff (Table 1) to gain insight into clinical decisions regarding the development of the role and each individual's involvement in the process.

**Table 1:** Staff interviewed and corresponding participant IDs

Participant ID	Role
S1	Head of Digital Solutions
S2	Head of People and OD Partnering
S3	Medical Secretary
S4	Administrative Support Assistant
S5	Head of Recovery and Planned Care Mental Health
S6	Clinical Director for Mental Health Services

ID, identification; OD, organisational development.

Finally, monthly in-depth interviews with the VCPs (Table 2) were conducted over 5 months. Each month, a different set of questions was asked, with an overarching theme. This enabled researchers to gain a deeper understanding of their motivations for applying and their experiences in the role over time. Given the nature of the position, all interviews were conducted virtually.

**Table 2:** Virtual psychiatrists interviewed and corresponding participant IDs

Participant ID	Role
VCP1	Older Adult Team and General Adult Virtual Consultant Psychiatrist
VCP2	Recovery Team and Crisis Resolution Home Treatment Team Virtual Consultant Psychiatrist
VCP3	Assessment to Intervention Team & Early Intervention in Psychosis Team Virtual Consultant Psychiatrist

ID, identification.

The content analysis and interview findings were used to create the step-by-step guide. The researchers collaborated on the guide with one of the successful applicants (N.F.N.), who is now 18 months into the role. N.F.N. contributed to the guide based on their own lived experience with the application process and working in the job. N.F.N. was not involved in the data collection or analysis, only in the writing of the guide.

The evaluation was co-designed between Solent NHS Trust and TEC Cymru. TEC Cymru is an all-Wales digital healthcare service funded by the Welsh government and based within Aneurin Bevan University Health Board. Solent NHS Trust obtained service evaluation approval, and TEC Cymru was provided with honorary contracts to conduct the evaluation. Verbal consent was obtained from all interviewees.

## Results

**Fig. 1:**
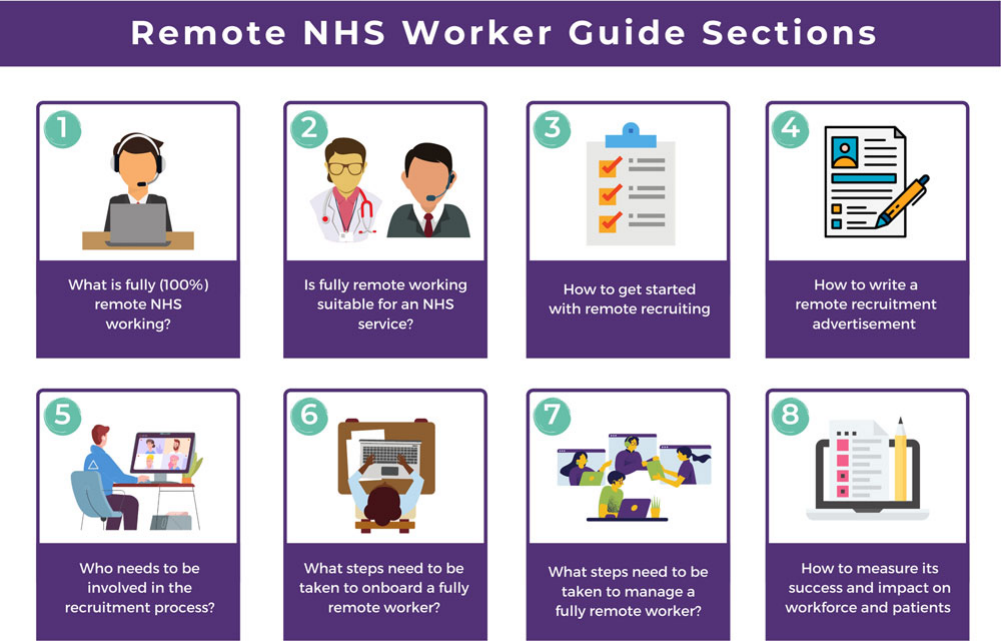
Contents of step-by-step guide.

The remote working guide is set out in eight sections (Fig. 1).

### Section 1: What is fully (100%) remote NHS working?

Virtual or remote working implies that someone can work from home, or close to home, using modern technologies. Remote working is frequently designed on a hybrid working approach, which allows some of the role to be performed at home and other parts in the office, community or clinical setting. Conversely, a fully (100%) remote worker is someone who has been hired for an entirely remote job that allows them to perform all work-related roles and responsibilities remotely. In a clinical role, this would include all patient care delivery, as well as additional job requirements such as training, supervision and research. Their contract would have to reflect all of this.

### Section 2: Is fully remote working suitable for an NHS service?

During their interviews, all three VCPs mentioned how the flexibility and accessibility of the role appealed to them.

‘*Can be anywhere, and support family when needed*’ – VCP2

Fully remote working provides more opportunities than hybrid approaches. It removes all geographic boundaries, allowing anyone from ‘anywhere in the world’ to apply for the job provided they have the appropriate qualifications, skills and experience. For the NHS, the removal of this boundary can significantly improve the opportunity for recruitment of staff to a broader audience with a much wider array of skills and expertise. This was a major benefit suggested by many of the recruitment and support staff. The VCP role has significantly improved the demand on the service, relieving current staff pressures and workloads. This sentiment was shared in one of the social media posts identified in the content analysis:

‘*Would rather have a colleague working 100% virtually than an unfulfilled vacancy’* – social media post

S1 believed that this new role demonstrates that the NHS is ‘catching up’ with other organisations that offer a more hybrid approach to working. Furthermore, S6 revealed that cost was a factor in the creation of a virtual consultant role; as the expense of hiring locums increased, it became clear that hiring three substantive consultants would be more cost-effective.

One social media user commented on the suitability of hybrid versus fully remote working.

‘*I think it risks excluding significant numbers of already digitally excluded and otherwise disadvantaged patients from treatment. Offer a combination of virtual and face-to-face for sure’* – social media post

This insight suggests that fully remote services may not be suitable; however, this does not preclude a specific physician's role from being fully virtual, provided the entire service is not. For example, Mental Health Act (MHA) assessments cannot be done virtually, so it is still important to consider non-virtual support. During a virtual clinic, if the VCP deems a patient to require a MHA assessment, they can refer to the crisis resolution home treatment team, who handle admissions and can conduct in-person assessments. The service also runs a well-being clinic, where all patients (including those who normally attend in-person psychiatric consultations) attend in person to have any hands-on physical health checks and baseline investigations.

Virtual roles do not have to be purely clinical; all three VCPs have supervisory and teaching roles, in addition to participating in research and innovation, just like many non-virtual consultants. Except for the MHA caveat, their role is the same as that of their in-person consultant psychiatrist colleagues. Not all NHS roles are suitable for fully remote working, with cleaners and surgeons being the most obvious examples, as their in-person presence is required for their job. However, many NHS roles can be performed remotely, and entirely remotely too. With advances in technologies such as video consulting and remote monitoring, clinicians can work virtually to provide robust clinical care.

### Section 3: How to get started with remote recruiting

To start the process of recruiting virtual staff, it is important to identify appropriate services and roles that can be performed fully remotely, as discussed above. The team in which such a role has been identified should be included in the initial conversations so they can meaningfully contribute to the process and understand the nature of the remote role.

Writing the job description may not require many differences compared with the equivalent for non-virtual jobs. S2 reported that when creating the new job description, only minor changes were required to incorporate the virtual aspect.

### Section 4: How to write a remote recruitment advertisement

The content analysis of applications identified common terms used by applicants to express why they felt they were best suited for the fully remote job. These terms could be reused for other virtual jobs to target the most suitable candidates. They included interest in the digitalisation of the NHS, implementing change, ambition, being a risk taker and flexibility.

In the interviews, the hired virtual psychiatrists were asked their reasons for applying for the job:

‘*Personal circumstances were the biggest factor, family matters more than money’* – VCP2

‘*Wanted to explore something new, thirsty for change’* – VCP3

Eleven of the 16 applicants mentioned prior virtual experience. However, VCP2 and VCP3 had relatively limited virtual work experience. This suggests the importance of emphasising in advertisements that virtual experience is not required, only an enthusiasm for the advancement of it.

All applicants had prior NHS work experience, providing an advantageous fundamental understanding of NHS infrastructure which could be applied when working virtually. Three were applying for their first consultant position, whereas the remaining 13 had previously worked as consultants for varying lengths of time. Experienced consultants can offer a deep understanding of NHS infrastructure and established processes, facilitating a smooth transition to online work. However, they may be less adaptable to change. By contrast, new consultants may bring fresh perspectives and adaptability but may lack experience in navigating existing systems. The key to successful virtual work lies not in experience alone but in finding an individual with the right mindset.

VCP1 and VCP2 saw the role advertised in the *BMJ*, whereas VCP3 saw it on Twitter. This shows the benefit of considering non-traditional places to advertise for these jobs, such as social media. VCP3 was not actively looking for a job so would not have seen the advertisement otherwise.

### Section 5: Who needs to be involved in the recruitment process?

Teams and individuals that should be involved include human resources, IT, communications, clinicians working in the department, a cybersecurity manager, a data protection officer and a senior information risk owner. Owing to the non-traditional nature of the role, a project manager is recommended to keep the jobs to a specific timescale to be delivered within expectations.

‘*Next time, we would get a project manager. This would enable us stick to time better alongside day jobs to prevent turning things round so quickly where individuals would be starting before contracts finalised’* – S2

‘*It all worked out because there was a strict timeline for the team to work towards, despite the challenges encountered’* – S6

### Section 6: What steps need to be taken to onboard a fully remote worker?

Successful onboarding is vital for both the organisation and employees. Although remote onboarding is feasible, in-person attendance may be beneficial for equipment collection, meeting key individuals and team integration. Regardless of the approach, introducing all team members and roles is crucial. Remote workers must be provided by the employer with equipment such as laptops, Dictaphones and headsets, including a variety to allow for personal preference and the use of alternatives when technical challenges arise. Formal training on all systems is necessary, along with contact details for specialised technological support personnel. Clear arrangements for replacing faulty equipment must also be in place. S4 commented that when something goes wrong with IT, getting support can be difficult and time-consuming owing to the process of shipping the equipment back and forth. This is unavoidable if working overseas, so employers should encourage remote workers to report any issues as soon as possible so they can be addressed.

Security considerations are critical; ensure any local or governance issues are resolved before the remote worker begins, especially if they are working outside the UK. Working abroad necessitates a cybersecurity assessment of the country in which the person wishes to work. Furthermore, when working from a remote location, VPN access is required to gain network access to ensure the Trust's network is secure and encrypted. Individual practitioners will need to seek tax advice based on the proportion of their time spent in the UK versus abroad. All VCPs were able to arrange insurance with a licence to practise in the UK, confirming their individual circumstances with insurers. A remote working policy should be in place and reviewed on a regular basis to account for significant changes in technology and regulation.

### Section 7: What steps need to be taken to manage a fully remote worker?

Working entirely remotely poses the risk of the clinician not feeling fully integrated within the team, so it is important to have technology in place to facilitate inclusion in all team, educational and other meetings. Regular check-ins, strong, consistent communication with the line manager, and adequate administrative support are also required when managing these workers. In Solent, there is currently one medical secretary working between the three VCPs, but adding more virtual roles would increase the administrative workforce.

The management of virtual workers does not need to be any different from that of their in-person colleagues; it simply requires a different mode of communication. Robust communication with fully remote clinicians (e.g. job plans, appraisals) is possible and effective for addressing issues and providing support. Licensing of clinical practice and professional development is in place and in keeping with General Medical Council and Royal College standards, as for all other consultant psychiatrists. VCPs are expected to participate in professional development in the usual way and have an annual appraisal like their in-person colleagues.

### Section 8: How to measure its success and impact on workforce and patients

With this being a novel concept, constant evaluation of remote working is vital. Audits, service evaluations and quality improvement projects are encouraged to compare the success and impact on patients of fully remote compared with non-fully remote working. Suggested ways to measure this impact include clinical outcomes, patient-reported outcome measures, staff surveys and service ratings.

## Discussion

Overall, the data from the content analysis, support staff interviews and VCP interviews helped to consolidate an understanding of the new role, allowing the creation of this step-by-step guide to remote working.

This study shows that fully remote psychiatrists can work well and safely within multidisciplinary NHS teams. There have been no adverse outcomes noted while the three VCPs in the Solent NHS Trust have been in post. This is supported by the NHS benchmarking data,^23^ offering insights into Solent NHS Trust's positioning in key metrics compared with other UK organisations during the time the VCPs have been in post. Moreover, previous randomised control trials found no difference in patient outcomes, follow-up or patient satisfaction between telepsychiatry and in-person care.^24,25^ These studies did not consider clinicians working entirely remotely, instead focusing on remote patients, but our study suggests that this is translatable to fully remote work. Equally, patients should always have the choice to be seen in person if they would prefer, in keeping with the ethics of patient autonomy.^14,26^ The benchmarking data show that Solent continues to maintain a good proportion of face-to-face contacts across adult mental health services, and this figure has increased compared with the previous year. This proves that digital methods are not replacing face-to-face interactions and highlights Solent's successful efforts in maintaining a balanced approach. Benchmarking has also revealed that Solent has lower overall costs than other organisations, supporting the cost-effectiveness of virtual hiring by employing substantive consultants rather than locums.^23^

Selecting the right NHS roles for fully remote working is critical to success. Being less of a hands-on specialty, psychiatry may be particularly well suited.^20^ Job advertisements must transparently communicate the virtual aspect but emphasise that fully remote jobs share the same expectations and responsibilities as in-person roles. They are simply executed virtually. However, there may be some exceptions where virtual workers cannot carry out all the same responsibilities as their in-person counterparts. These must be recognised proactively and excluded from job descriptions. Although necessary during the pandemic, remote MHA assessments pose an ethical dilemma within telepsychiatry.^27,28^ In 2021, the law changed to require all healthcare professionals conducting an MHA assessment to be physically present, placing a limitation on fully remote consultant psychiatrists.^29^ This demonstrates why it is important to have the full support of the clinical team when establishing virtual roles, as in-person support is still necessary for their success. It is important to note that although psychiatry is a generally hands-off specialty, there may still be a need for hands-on examination, such as monitoring for drug side-effects, again demonstrating a limitation of virtual consulting and the importance of in-person support from the team.

All other aspects of the role including clinical work, teaching, training, research and innovation have been demonstrated to be possible by the VCPs. Furthermore, although VCPs cannot perform MHA assessments, they could be better positioned to identify those patients who require assessment, as the most ill patients may find it difficult to leave their homes and attend in-person appointments but might attend virtual appointments.

Communication is critical to the success of remote working, as demonstrated across most job industries during the pandemic.^30^ The importance of communication has been researched when leading agile workers in the NHS,^31^ as well as when managing short-term entirely remote NHS teams during the pandemic,^32^ and this can also be applied to permanent fully remote workers. Virtual healthcare professionals, like other remote workers, require continuous organisational support. Working from home can affect individuals’ mental well-being and cause feelings of isolation.^33-35^ Regular check-ins are crucial, with an emphasis on prioritising health, taking screen breaks, moving and staying hydrated.

Regarding the IT aspect of the role, cybersecurity is essential owing to the sensitive and confidential nature of healthcare records,^36^ particularly if individuals are working from abroad. Remote workers must be educated around staying cybersafe to minimise disruptions in access to trust systems which could affect patient care. Adequate technology, training and technical support are vital to promptly address any issues that may arise, as well as ensuring that clinicians can adequately document everything for medico-legal purposes.^28^

The existing literature tends to discuss the process of hiring hybrid NHS workers, which is entirely different to that required for fully remote work. The Solent NHS Trust experience emphasises the importance of effective project management in the recruitment and retention of fully remote staff, especially for organisations venturing into this for the first time.

In conclusion, fully remote working as a consultant psychiatrist in the NHS is feasible and effective, with its applicability extending to various healthcare roles and specialties. However, when thinking about virtual recruitment, careful consideration is needed to identify appropriate roles in suitable, open-minded teams. Ongoing evaluation of the impact on patients and clinicians is also essential. Sharing insights within and beyond the organisation has led to the expansion of fully remote roles in the Solent NHS Trust. More remote clinicians have now been recruited, as the initial scheme worked well and has informed the successful hiring of remote nurse practitioners working in community adult mental health. Although no apparent differences in outcomes or adverse events have been observed, a formal comparison of patient outcomes from virtual and remote psychiatrists could be beneficial. Moreover, although completely remote roles are possible, global patient services should remain hybrid, and patients should ultimately have a choice between in-person and virtual appointments. Future research could investigate the impact of virtual supervisors on trainee supervision and training.

## Data Availability

The participants of this study did not give consent for their data to be shared publicly, so interview transcripts and job applications are not available.

## About the authors

**Megan Havard** is a research assistant at Technology Enabled Care Cymru, Aneurin Bevan University Health Board, Newport, UK. **Nyembezi Faith Ndebele** is a consultant psychiatrist at Solent NHS Trust, Southampton, UK. **Suyog Dhakras** is a consultant child and adolescent psychiatrist at Solent NHS Trust, Southampton, UK. **Gemma Johns** is Head of Research at Technology Enabled Care Cymru, Aneurin Bevan University Health Board, Newport, UK. **Ian McCafferty** is a consultant psychiatrist and Clinical Director at Solent NHS Trust, Southampton, UK. **Alka Ahuja** is National Clinical Lead at Technology Enabled Care Cymru, Aneurin Bevan University Health Board, Newport, UK.
